# Healthcare Professionals’ Perceptions of Promoting Healthy Lifestyle Behaviors in Pregnant Migrant Women and the Potential of a Digital Support Tool—A Qualitative Study

**DOI:** 10.3390/ijerph19042328

**Published:** 2022-02-17

**Authors:** Emmie Söderström, Ulrika Müssener, Mikaela Löfgren, Linnea Sandell, Kristin Thomas, Marie Löf

**Affiliations:** 1 Department of Health, Medicine and Caring Sciences, Division of Society and Health, Linköping University, 581 83 Linköping, Sweden; ulrika.mussener@liu.se (U.M.); mikaela.lofgren@live.se (M.L.); linsa940@student.liu.se (L.S.); kristin.thomas@liu.se (K.T.); marie.lof@ki.se (M.L.); 2 Group MLÖ, Department of Biosciences and Nutrition, Karolinska Institutet, NEO, 141 83 Huddinge, Sweden

**Keywords:** mHealth, telemedicine, migrant, healthcare, maternity care, pregnancy, health behavior, qualitative research methods

## Abstract

Eating healthily and being physically active during pregnancy are important for maternal and offspring health. Maternity healthcare is a key arena for health promotion; however, 20% of pregnant women in Sweden are foreign-born, which may reduce reach due to language and cultural barriers. The aims of this study were to explore healthcare professionals’ perceptions about (a) promoting health behaviors (i.e., healthy diet, physical activity, and weight gain) among Arabic- and Somali-speaking pregnant women and (b) how a translated version of the previously evaluated Swedish app (HealthyMoms) can be tailored and used as a tool in their clinical work. Healthcare professionals in Swedish maternity care (*n* = 14) were interviewed. Data were analyzed using inductive thematic analysis. Healthcare professionals expressed challenges in health promotion work, including cultural and educational aspects and low awareness of health behaviors among women themselves and their social environment. Further, a lack of resources within the clinical practice and a need for cultural awareness among healthcare professionals were highlighted. Finally, it was perceived that a translated app has potential to provide basic and culturally adjusted information, facilitate communication and thus has potential to become a helpful tool in maternity care to support healthy lifestyle behaviors in Arabic- and Somali-speaking pregnant women.

## 1. Introduction

A healthy lifestyle during pregnancy is important for optimal health in both mother and offspring. One important aspect of a healthy pregnancy is gestational weight gain (GWG), as both excessive and inadequate GWG are associated with negative health outcomes [1]. For instance, excessive GWG has been associated with a higher risk of caesarian delivery, large-for-gestational-age infants, pre-eclampsia, and gestational diabetes [2,3], whereas inadequate GWG has been associated with, e.g., small-for-gestational-age infants and preterm delivery [4,5]. In Sweden, approximately 50% of pregnant women exceed the GWG recommendations and 20% have inadequate GWG [4,6]. Excessive and inadequate GWG is also prevalent in migrant women in Sweden and Swedish registry data have shown that migrant women have an increased risk for both inadequate and excessive GWG compared to native women [6]. Additionally, migrant women have higher risks of adverse pregnancy outcomes, including maternal mortality and pre-term birth compared to native women [7]. Furthermore, migrant women have a higher prevalence of gestational diabetes [8], which is concerning given the higher risk for pregnancy complications as well as the increased risk of type 2 diabetes and cardiovascular disease within 10 years after delivery [9,10]. Considering the high prevalence of excessive and inadequate GWG among the population and the associated adverse pregnancy outcomes, maternity healthcare is a key arena for promoting a healthy lifestyle during pregnancy. Given the inequalities referred to above, particular emphasis on migrant women is important to make sure that this work is inclusive for all women, irrespectively of language and cultural context.

Previous lifestyle interventions promoting healthy eating and physical activity behaviors provided within the healthcare setting have shown to be effective in reducing excessive GWG [11]. However, these interventions have been delivered in person and may be demanding for healthcare to scale up considering costs and personnel resources [12], especially if there are language barriers and interpreters are required. Another approach is to deliver interventions using mobile health (mHealth), i.e., mobile technology as a support for health [13], which has the potential to use less resources compared to traditional interventions, while reaching more people [12]. We recently developed a novel mHealth intervention delivered through a smartphone app in pregnant women (the HealthyMoms app) [14]. Our previous work when evaluating this app both in quantitative and qualitative studies have shown that users perceived the app to be trustworthy with relevant content for supporting health behaviors [15] and that the app had beneficial effects on GWG in women with pre-pregnancy overweight and obesity in the intervention group (−1.67 kg [95% CI −3.26; −0.09]) versus the control group that received standard care. Furthermore, the intervention group had better dietary habits at follow-up compared to the control group [14]. The effect of the HealthyMoms intervention on GWG was similar to previous interventions delivered in person [16], highlighting the potential of scaling up the HealthyMoms intervention to reach more pregnant women across Sweden. However, the app is currently only available in Swedish, which limits the reach since 20% of women attending maternity healthcare in Sweden are foreign-born [6].

Previous research has indicated that technology-, health literacy-, and language difficulties could affect the uptake of pregnancy interventions in migrant women [17]. Therefore, it is important to not only translate existing mHealth tools but also to tailor and make relevant modifications to ascertain reach and usage. Since a majority of migrants in Sweden come from Iraq, Syria, and Somalia [18], a first relevant step of this work would be to translate the app into Somali and Arabic and to make relevant cultural adaptations. We are currently conducting a research project to tailor the HealthyMoms app and evaluate it as a tool to promote healthy lifestyles behaviors among Arabic- and Somali-speaking pregnant women in maternity healthcare. The project is a part of the Mobile health Multiple lifestyle Behavior Interventions across the LifEspan (MoBILE) research program, which includes seven randomized controlled trials in different patient groups [19]. In order to understand how the HealthyMoms app could best be adapted and used within maternity healthcare, it is necessary to get insight from healthcare professionals that frequently meet and work with health behaviors in migrant women. Therefore, the specific aims of this qualitative study were to explore healthcare professionals’ perceptions about (a) promoting health behaviors (i.e., healthy diet, physical activity, and weight gain) among Arabic- and Somali-speaking pregnant women and (b) how a translated version of the previously evaluated Swedish app (HealthyMoms) can be tailored and used as a tool in their clinical work.

## 2. Materials and Methods

### 2.1. Study Design

This study employed a qualitative design [20] with individual interviews. Inductive thematic analysis was performed [21]. The study was carried out according to the COnsolidated criteria for REporting Qualitative research (COREQ) checklist (Scheme S1) [22].

### 2.2. Informants and Recruitment

A purposive sampling [20] of healthcare professionals working within maternity healthcare was used. The informants were recruited by email from the maternity and endocrinology clinic as well as the delivery ward at Linköping University Hospital, Sweden. Inclusion criteria were healthcare professionals that regularly meet pregnant migrant women in their practice and that were willing to participate in an interview. Informants were recruited between May 2020 and June 2021. In total, 14 healthcare professionals, one male and 13 females, reported interest in participating and a time for an interview was scheduled. Informants represented a variety of professions, including midwives (*n* = 10), physicians (*n* = 3), and a dietician (*n* = 1). The mean age was 49.8 (SD 10.3) years and informants had on average 18.1 (SD 12.6) years of work experience. The majority were born in Sweden (*n* = 11), or in Romania, Germany, or Poland (*n* = 3).

### 2.3. Ethics

This study was conducted in accordance with the Declaration of Helsinki. The study received ethical approval by the Swedish Ethical Review Authority (Reference number: 2020-01447). All participants provided their written informed consent prior to interviews.

### 2.4. Data Collection

A semi-structured interview guide was developed by the research team with expertise in pregnancy, nutrition, physical exercise, health psychology, qualitative- and mHealth research. The guide consisted of a set of main questions covering current working routines related to encouraging health behaviors among migrant women within maternity healthcare, how a smartphone app could be used to support a healthy lifestyle, and how the HealthyMoms app could be modified to reach this specific group. Probing questions were asked based on individual responses.

At the time for the interview, informants were provided with verbal information about the study and that they could withdraw at any timepoint. Prior to each interview, written informed consents were collected and informants answered a short questionnaire (workplace, health profession, and years of work experience with pregnant women). Due to the COVID-19 pandemic, informants could choose between having the interview in person (*n* = 11) or over telephone (*n* = 3). All in-person interviews were conducted at the workplace of the informant except for one interview that was conducted at Linköping University. Interviews were conducted by one interviewer, either by first author, a female nutritionist and PhD student with previous experience of conducting interviews for qualitative research and with basic education and training in qualitative methodology (ES) or by female research assistant and medical student with experience from maternity healthcare and pregnancy (MIL). Data collection was performed by ES and MIL in close collaboration with senior researchers with long experience of qualitative data collection and analysis. The interviews were audio recorded and lasted between 29–65 min (average 42 min). Field notes were taken during interviews. All interviews were transcribed verbatim by a transcription firm.

### 2.5. Data Analysis

**Figure 1:**
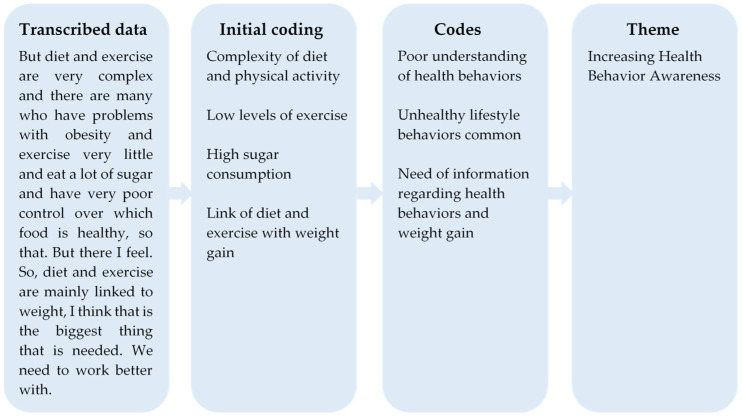
Illustration of the data analysis process from transcribed data to final theme.

Data were analyzed using inductive thematic analysis [21]. The following steps were performed: ES and female medical student LS familiarized themselves with interview data by listening to all interviews and reading transcripts several times. Thereafter, initial codes were identified in the transcribed data by ES and LS that coded half of the interviews each. The coding process was data driven and the codes were created in relation to the aim of the study and were developed during an iterative process. After individual coding by ES and LS, coding sheets were merged whereby, through discussions, consensus on a single coding sheet was reached. Thereafter, ES proceeded reviewing the coding of all the interviews. This process was supported with continuous discussion and validation by UM and KT (female researchers with expertise in qualitative methodology) who had read all interviews. After systematic coding of the whole dataset, ES searched for patterns in the data and sorted codes into possible themes. These themes were reviewed, revised, and refined by ES, UM, and KT until consensus was reached regarding their content and labelling. An example of the coding process is presented in Figure 1.

## 3. Results

**Figure 2:**
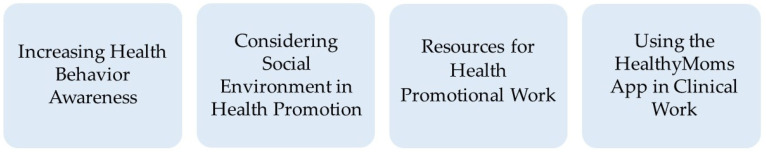
Themes from thematic analysis.

This interview study set out to explore healthcare professionals’ perceptions about promoting health behaviors among migrant pregnant women. The study also explored perceptions about using the HealthyMoms app in clinical work and how it can be adjusted to Arabic- and Somali-speaking pregnant women. Four themes were identified: (1) Increasing health behavior awareness; (2) Considering social environment in health promotion; (3) Resources for health promotional work; and (4) Using the HealthyMoms app in clinical work (Figure 2). Each theme is described below and includes interview excerpts to underpin data categorization. Quotations are presented along with informant characteristics and non-relevant sections for the quotations are denoted with […].

### 3.1. Increasing Health Behavior Awareness

Informants voiced that although migrant women make up a heterogenous group with varied needs and preferences, a key element of health promotion work is to raise patients’ awareness of pregnancy and bodily changes, providing guidelines for healthy diet and physical activity, as well as recommendations for GWG. Informants expressed raised awareness of health behaviors to be an important tool for behavior change, specifically in terms of dietary habits and physical activity. A concern among informants was that low levels of awareness could impose a health risk for migrant women by, e.g., limiting adherence to health recommendations or treatments for gestational diabetes.

*“I am very unsure if they are aware of… I think, for example, the National Food Administration has certain recommendations for pregnant women. I do not know if they are aware of them. If they have information about them and if they think they are relevant.”*

(Midwife, delivery ward, interview 10)

Informants described that women’s educational level and cultural background affect their needs and preferences for health information and influence how information is understood. For example, informants perceived educated women, in general, to have a good awareness of pregnancy-related health information, whereas reaching illiterate women was perceived to be more difficult as the health information currently available in maternity care is not tailored to this group. Moreover, stressful life events and uncertain living conditions, such as migration status or worry about the health of family and friends were described to make it more difficult for the women to focus on living healthy and being aware of health behaviors.

*“I can imagine that those who come from a country and have not come here voluntarily, may have come here as a refugee or have had to flee from their country, they do not really focus on things like this [lifestyle habits] that I think that they sometimes experience as a bit trivial. They have many other major issues in their lives that they need to solve. Maybe ‘can I stay at all?’, ‘Can my children come here?’, ‘Can my husband come here?’”*

(Midwife, maternity care, interview 12)

Cultural background and previous healthcare experiences in women’s native countries were described to contribute to the perceived need for care, including pregnancy monitoring. Informants further described that risk-awareness among migrant women regarding excessive or insufficient GWG or gestational diabetes, is limited and urged that there is a need for tailored health information and advice to prevent these outcomes.

*“I think you often can see that they do not care as much about how much weight they gain during a pregnancy and you can often see that the weight increases quite quickly and do not absorb the same information about healthy weight gain and healthy lifestyle habits. I think you can see a big difference there.”*

(Midwife, maternity care, interview 6)

### 3.2. Considering Social Environment in Health Promotion

Informants described the importance of considering the social environment of the pregnant woman when discussing health behaviors with them. Family and friends were often perceived to be engaged in pregnancies which could work both as a barrier for promoting healthy behaviors and a resource for change. For example, informants expressed concerns that women trust information from their social environment and cultural context to a greater extent than recommendations provided by healthcare authorities. Informants further highlighted the need to consider partners and families in their work as they have an essential role in motivating, encouraging, and reminding the women to, e.g., stay physically active and eat healthily. Partners’ worries were also expressed as something that the health professionals needed to address on a regular basis. A common perception was that partners are worried, for instance, due to a poor understanding of common pregnancy symptoms. It was also highlighted that partners might not realize the importance of healthy habits for the mother and fetus.

*“That you [migrant women] value experiences from previous generations higher than we do. […] It is important to convince the partner but also the women in the surrounding environment such as sisters, mothers, mother-in-law, that in some cases can play an important role. Possibly that we [healthcare professionals] should broaden our views there as well and also aim to direct the information to the women in the surrounding environment.”*

(Midwife, delivery ward, interview 10)

The informants felt obliged to address expectations from the social environment that hinder pregnant women from maintaining health behaviors, for example, misconceptions that pregnancy requires eating for two or that physical activity can harm the fetus. Similarly, expectations on women’s domestic roles were expressed as something that could hinder women from prioritizing health behaviors.

*“You [the women] sleep poorly, you eat poorly, you move too little, you have pretty much none, or almost no, hour of the day where you are alone. Without having to be present and available with your womb, with your body, with your household chores, with your breasts for the children.”*

(Midwife, maternity care, interview 9)

However, informants also discussed that women often have an important role in their social environment which was described as an opportunity for health promotion. For example, women are often the primary care giver and responsible for the eating habits of the whole household. By reaching pregnant women, their social environments were believed to also be influenced due to the central role women often play in the family setting.

*“But if the woman understands, it is still the woman who fixes the food at home… If the woman herself understands, it is a gain for the whole family, in fact. Both children and husband.”*

(Physician, endocrinological clinic, interview 8)

### 3.3. Resources for Health Promotional Work

The informants expressed that there is a need for increased resources to enable health promotion work and support Arabic- and Somali-speaking pregnant women. For example, an increased cultural awareness among healthcare professionals and more time in the meeting with migrant women were requested. It was further expressed that healthcare professionals need knowledge about common foods within various cultures to provide dietary recommendations. Similarly, there was a perception that cultural awareness is needed to give relevant recommendations for physical activity and provide support for a healthy GWG.

*”To rest on the experience, how it [cultural foods] usually is. And maybe go into details a little. […] So you need to have dug a lot more into the food culture to know what everything means here.”*

(Dietician, endocrinological clinic, interview 3)

Informants also expressed that building rapport and trust is essential to reach women and be able to succeed with health promotion work. However, cultural differences between themselves and the women were perceived to make trust building a challenge.

*“And then, there is very low trust in us. They do not trust us. And then they have a very hard time following our recommendations. […] Their trust in us is not very strong, so if we recommend medical treatment, it takes time until they understand or accept, so those are challenges.”*

(Physician, endocrinological clinic, interview 5)

Informants perceived that limited time in routine health visits generally challenges the work with health behaviors. It was further discussed that within the migrant group, it might be even more challenging as there are language barriers, diverse needs for health information, and misconceptions that need to be addressed, requiring more time than what is typically allocated for a routine health visit.

*”As I said before, this that you often feel like you could put more energy into these topics [health behaviors] if we had the time, which we do not have room for.”*

(Midwife, maternity care, interview 13)

Informants described that certified interpreters are available to minimize language barriers and increase healthcare professionals’ cultural awareness. However, the informants perceived that using interpreters in clinical practice complicates communication and makes conveying health information to migrant women more challenging. Specifically, it was perceived difficult to convey health messages adequately through a third party and informants expressed concerns that complex health information was simplified or lost in translation.

*“It can be difficult to reach through to them [the women] in a good way when using an interpreter. […] Difficult to get through with tools or tips that suit them and their culture or lifestyle. It may be difficult to motivate them.”*

(Midwife, maternity care, interview 6)

### 3.4. Using the HealthyMoms Application in Clinical Work

Healthcare professionals were asked to reflect on using a translated version of the HealthyMoms app in their clinical practice. Informants reported high usage of smartphones in the patient group and expressed that an app to support healthy lifestyle behaviors would be suitable for Arabic- and Somali-speaking women. Informants identified various benefits with the app, including that it would offer elementary and culturally adjusted health information which in turn could facilitate communication and offer support throughout pregnancy to both women and their families. Informants perceived that the app could potentially contribute to improved healthcare quality by reaching a culturally diverse target group. Health professionals working with gestational diabetes discussed that a culturally adapted app, with some added features, such as blood glucose monitoring and support for a healthy diet to manage this specific condition, could be incorporated into current routines and help ease the burden of healthcare considering the increased number of women getting diagnosed and the high prevalence among migrant women.

*“Yes, I think that our job automatically becomes easier when the woman has a higher level of knowledge and maybe a bit more compliance to good diet and exercise habits through this [the app]. It will feel easier for us to communicate about this. And hopefully that it then leads to that if we are really lucky, or what you say, and it works then the pregnant woman should gain less [weight], have lower risk of gestational diabetes and then that means less work for us, yes, like that. If one is to think about all the benefits.”*

(Midwife, maternity care, interview 2)

The informants discussed various clinical purposes of the app, for example being the main tool for health behavior promotion which could free up resources for patients with greater care needs. In contrast, the app was also perceived as a complement to current work routines since the physical meeting was thought to still be central in clinical work.

*“I still think we need to keep it [the health promotion work] a bit in the profession and bring in effective aids. But it cannot be replaced with just an IT solution, you must have both. I feel that. So I feel that there is a risk that it will only be digitized. I think that’s a shame. You must have aids, but still need the skills of midwives and be able to talk about these things. I think that is important from a public health perspective.”*

(Physician, maternity care, interview 14)

Although linguistic translation alone was perceived to benefit clinical work, the informants expressed that a more comprehensive cultural adaptation of the app would enhance relevance and usability for the women. Such adaptations include making the app inclusive and recommending foods and physical activities that are culturally relevant. Finally, the informants agreed that the app needs to be simple and include basic health-related information using different media types, such as text, video, and audio.

*“But the starting point must be in their own context. What are their lifestyles? What knowledge have they gained from growing up? How should we think now? What was good, what was not good?”*

(Midwife, maternity care, interview 12)

## 4. Discussion

### 4.1. Main Findings

Our findings demonstrated that healthcare professionals experience various challenges with health promotion work in maternity care, including limited awareness of health behaviors among migrant women themselves and in their social environment. Further, health promotion work was perceived to be influenced by patients’ education, culture, trust, and previous experiences. Additionally, lack of time in the healthcare practice and lack of cultural awareness in healthcare professionals could influence health promotional work. Moreover, informants perceived that a translated and culturally adjusted app has potential to be a helpful tool in promoting health behaviors in migrant women.

### 4.2. Comparison with Previous Work

To the best of our knowledge, this is the first study to explore perceptions among healthcare professionals on promoting health behaviors in Arabic- and Somali-speaking pregnant women within maternity care in Sweden. Further, it is the first study to obtain insight on how an app could be adjusted to best support health behaviors in migrant pregnant women in a Swedish setting. We identified several factors that influence how health information is adopted, e.g., education, previous experience, culture, and migration status. These findings are comparable to results from a systematic review that found that diet as well as physical activity in women migrating from African countries to high-income countries (age: 16–67 years) were influenced by cultural, religious, and migration aspects, as well as their perceptions about health [23]. Moreover, a Swedish qualitative study in non-pregnant Somali women showed that misconceptions related to physical activity behaviors depended on previous experiences and traditions [24], which was also seen in our data. Our results further demonstrated that women’s social environment could be a valuable support for healthy lifestyle behaviors given that family and friends often have a large involvement during pregnancy. However, informants also expressed that the social environment could be a barrier, since advice from family and friends might be contradictive to national guidelines provided by healthcare. Our findings align with a comparable study in Pakistani women who had migrated to the U.K., where healthcare professionals perceived that family has an important role but could, if too dominant, have a negative impact on the relationship between healthcare professionals and patients in maternity care [25]. Furthermore, our results demonstrated that informants perceived the patient group to lack trust in their healthcare provider. Similar findings have been reported in other healthcare settings [26], indicating that there is a need for further research to understand how trust and communication can be improved in order to disseminate recommendations by healthcare professionals in a trustworthy way. To conclude, our findings show that both external and internal factors are important to consider when working with health promotion in pregnant migrant women, and that special emphasis should be put on the role of the social environment.

We also identified resources needed in maternity care to promote health behaviors, including need of cultural awareness. Increased cultural competence has previously been identified as a need in the healthcare meeting for healthcare professionals working with pregnant migrant women and migrant families with young children [27]. Additionally, we found that resources, such as satisfying communication, time, and trust are essential in the health-promoting meeting. A previous review by Kasper et al. [28] focusing on maternity healthcare in general and not specifically health promotion work, found that communication was perceived as a barrier within maternity care [28], which is in line with our findings. We also found that available resources within the care setting, i.e., interpreters, were sometimes believed to negatively influence communication in the health promoting meeting. Even though interpreters are available as a valuable support acting as a bridge in the communication between the patient and healthcare provider, it may be challenging to ascertain that no vital information is lost or modified in the patient meeting.

We found that informants perceived awareness of health behaviors to be generally low in the patient group and that the HealthyMoms app could be a way to overcome this by providing basic health information in the women’s native languages. Similarly, Mårtensson et al. [29] found that Somali and Arabic migrants in Sweden expressed needs of receiving comprehendible health information from healthcare. Furthermore, there was an expressed need among migrants for cultural awareness among healthcare professionals, which informants in our study also emphasized. A similar finding was presented by Kasper et al. [28], highlighting the need of transcultural competence in healthcare professionals, to provide individualized care.

Previous studies on translating and adapting mHealth interventions to target migrant populations are scarce. However, our research group is conducting similar work for child healthcare as for maternity healthcare [30]. We have recently published a qualitative study [31] where we investigated insights on how a Swedish app for parents of pre-school aged children could be developed to reach Arabic- and Somali-speaking parents. In accordance with our findings in this study, healthcare professionals in child healthcare proposed that graphical content, i.e., audio- and video-files could be beneficial to include in the app in order to reach parents with low levels of literacy.

### 4.3. Strengths and Limitations

This study has several strengths, including the comprehensive data from individual interviews with healthcare professionals that had experience from working with migrant women. The qualitative approach [20] provided deep and rich data of the phenomena studied, i.e., experiences of health promotional work in migrant women and how a translated app could be used in maternity care. Informants were recruited purposefully and included various professions (midwives, physicians, and a dietician) from different clinical practices (maternity care, delivery ward, and an endocrinology clinic) as well as a broad range of work experience and ages, which further contributed to broad and rich data [32]. Moreover, the sample included informants born outside of Sweden which could provide meaningful insights about the phenomena. Recruitment was carried out until the data material was assessed to adequately capture the study aims in terms of richness and depth. Hence, data collection was finalized when saturation was reached, i.e., when the researchers assessed that rich and broad data had been collected to ensure that the research aim could be answered [20]. This study was limited by the fact that the interviews focused on migrant women speaking Arabic and Somali and thus we cannot transfer our findings to other migrant populations. However, a majority of migrants in Sweden come from Iraq, Syria, and Somalia, i.e., Arabic- and Somali-speaking countries [18], which is the reason for our choice to start tailoring the app for these groups. Translating the app into Somali and Arabic will increase our reach of the app and substantially support the promotion of health behaviors in maternity healthcare throughout Sweden. Nevertheless, this should be considered as a first step, and we aim to include more languages in future versions of the app if requested.

To enhance trustworthiness of the results [20], several strategies were used. First, dependability was increased through the use of an interview guide that enabled a structured and systematic data collection. Further, data analysis followed a thematic analysis method according to the steps recommended by Braun and Clarke [21], which contributed to a rigorous and systematic process. Credibility was further strengthened [33] in data analysis through investigator triangulation given that data was initially independently coded by two authors and the resulting themes were then discussed thoroughly within the research team. An additional strength is the engagement of researchers with various backgrounds and expertise that systematically analyzed the data. Representative quotations from the transcribed texts were presented to further increase credibility. Regarding transferability, the study was conducted within a healthcare setting in Sweden including informants that regularly meet migrant pregnant women. The informants had various professions and working life experiences, which likely resemble other healthcare workers in similar healthcare settings in Sweden. In this study, we have strived to give a rich description of the study procedure, setting, and informants which could contribute to an increased transferability of the results [33].

### 4.4. Implications and Clinical Relevance

This study provides important insights and views from healthcare professionals on how to best support health behaviors in migrant women during pregnancy. One important finding was that increased cultural knowledge among healthcare professionals would be beneficial to support health behaviors in migrant women. Therefore, it might be relevant to increase such knowledge among healthcare professionals within maternity care to provide relevant recommendations that are suitable for migrant women. Our results also indicate that there is an interest for digital tools in maternity care to promote health behaviors and that a translated and adapted version of the HealthyMoms app has the potential to provide patients with necessary information about health behaviors in pregnancy. Interestingly, a translated app in Arabic and Somali was also seen as a particularly valuable tool by healthcare professional working with gestational diabetes, as traditional care with in-person and telephone counselling is challenging for healthcare due to the fact that the prevalence of gestational diabetes has increased during recent years [34] and is high among migrant women [8]. Moreover, in this context it is relevant to note that smartphone access in the Swedish population is high (98% in the ages 25–34) [35]. Our informants also expressed that access to technology is not an issue in our target population. Hence, interventions delivered through smartphones are likely to reach migrant pregnant women in Sweden and have additional advantages, including that information can be easily updated, tailored, and provided in various languages. Furthermore, our results indicate that the app could be a resourceful tool in the patient meeting, especially since informants expressed that interpreters might influence the information that is provided to the patients. Thereby, the app could be a resource in the health-promoting meeting. To fully understand what support is needed and how the app could be best adapted to reach as many women as possible, insights from the end-users, i.e., the women themselves, are needed. We are currently conducting interviews with Arabic- and Somali-speaking women to complement this study’s findings. Thereafter, the app will be translated and adapted before being tested in a pilot randomized controlled trial [19].

Given that informants in our study expressed that pregnant migrant women have low trust in healthcare professionals, these findings need to be verified in the patient group as well. Additionally, further research needs to address whether misunderstandings in how healthcare recommendations are perceived differ between native Swedish women and migrant women, and if so, how dissemination by healthcare professionals can be improved to counteract potential health inequalities.

## 5. Conclusions

Healthcare professionals identified several factors that influence health behaviors in migrant women, including educational level, language, culture, and social environment. Healthcare professionals’ limited cultural awareness and insufficient resources in their practice were further perceived to affect health promotional work among this group. An adjusted and translated version of the HealthyMoms app was seen as a tool with potential to support health behaviors for both healthcare professionals within maternity care and Arabic- and Somali-speaking pregnant women.

## Data Availability

The data analyzed in this study is not publicly available due to restrictions in the ethical approval according to our national ethical guidelines, but are available from the corresponding author on reasonable request.

## Supplementary Materials

The following supporting information can be downloaded at: https://www.mdpi.com/article/10.3390/ijerph19042328/s1, Scheme S1: COnsolidated criteria for REporting Qualitative research (COREQ) checklist.

## References

[1] Voerman E., Santos S., Inskip H., Amiano P., Barros H., Charles M.A., Chatzi L., Chrousos G.P., Corpeleijn E., Crozier S. (2019). Association of gestational weight gain with adverse maternal and infant outcomes. JAMA.

[2] Goldstein R.F., Abell S.K., Ranasinha S., Misso M., Boyle J.A., Black M.H., Li N., Hu G., Corrado F., Rode L. (2017). Association of gestational weight gain with maternal and infant outcomes: A systematic review and meta-analysis. JAMA.

[3] Santos S., Voerman E., Amiano P., Barros H., Beilin L.J., Bergström A., Charles M.A., Chatzi L., Chevrier C., Chrousos G.P. (2019). Impact of maternal body mass index and gestational weight gain on pregnancy complications: An individual participant data meta-analysis of European, North American and Australian cohorts. BJOG Int. J. Obstet. Gynaecol..

[4] Goldstein R.F., Abell S.K., Ranasinha S., Misso M.L., Boyle J.A., Harrison C.L., Black M.H., Li N., Hu G., Corrado F. (2018). Gestational weight gain across continents and ethnicity: Systematic review and meta-analysis of maternal and infant outcomes in more than one million women. BMC Med..

[5] Han Z., Lutsiv O., Mulla S., Rosen A., Beyene J., McDonald S.D. (2011). Low gestational weight gain and the risk of preterm birth and low birthweight: A systematic review and meta-analyses. Acta Obstet. Gynecol. Scand..

[6] Henriksson P., Sandborg J., Blomberg M., Nowicka P., Petersson K., Bendtsen M., Rosell M., Löf M. (2020). Body mass index and gestational weight gain in migrant women by birth regions compared with Swedish-born women: A registry linkage study of 0.5 million pregnancies. PLoS ONE.

[7] Heslehurst N., Brown H., Pemu A., Coleman H., Rankin J. (2018). Perinatal health outcomes and care among asylum seekers and refugees: A systematic review of systematic reviews. BMC Med..

[8] Behboudi-Gandevani S., Parajuli R., Vaismoradi M. (2021). A systematic review of the prevalence of gestational diabetes in Norway. Int. J. Environ. Res. Public Health.

[9] Song C., Lyu Y., Li C., Liu P., Li J., Ma R.C., Yang X. (2018). Long-term risk of diabetes in women at varying durations after gestational diabetes: A systematic review and meta-analysis with more than 2 million women. Obes. Rev..

[10] Kramer C.K., Campbell S., Retnakaran R. (2019). Gestational diabetes and the risk of cardiovascular disease in women: A systematic review and meta-analysis. Diabetologia.

[11] Muktabhant B., Lawrie T.A., Lumbiganon P., Laopaiboon M. (2015). Diet or exercise, or both, for preventing excessive weight gain in pregnancy. Cochrane Database Syst. Rev..

[12] O’Brien O.A., McCarthy M., Gibney E.R., McAuliffe F.M. (2014). Technology-supported dietary and lifestyle interventions in healthy pregnant women: A systematic review. Eur. J. Clin. Nutr..

[13] The World Health Organization mHealth: Use of Appropriate Digital Technologies for Public Health: Report by the Director-General. https://apps.who.int/iris/handle/10665/274134.

[14] Sandborg J., Söderström E., Henriksson P., Bendtsen M., Henström M., Leppänen M.H., Maddison R., Migueles J.H., Blomberg M., Löf M. (2021). Effectiveness of a Smartphone App to Promote Healthy Weight Gain, Diet, and Physical Activity During Pregnancy (HealthyMoms): Randomized Controlled Trial. JMIR mHealth uHealth.

[15] Sandborg J., Henriksson P., Larsen E., Lindqvist A.-K., Rutberg S., Söderström E., Maddison R., Löf M. (2021). Participants’ Engagement and Satisfaction with a Smartphone App Intended to Support Healthy Weight Gain, Diet, and Physical Activity During Pregnancy: Qualitative Study Within the HealthyMoms Trial. JMIR mHealth uHealth.

[16] Shieh C., Cullen D.L., Pike C., Pressler S.J. (2018). Intervention strategies for preventing excessive gestational weight gain: Systematic review and meta-analysis. Obes. Rev..

[17] Hughson J.P., Daly J.O., Woodward-Kron R., Hajek J., Story D. (2018). The rise of pregnancy apps and the implications for culturally and linguistically diverse women: Narrative review. JMIR mHealth uHealth.

[18] Statistics Sweden Number of Migrations and Migrants during the Period 2010–2019 by Country of Birth and Number of Migrations. https://www.scb.se/en/finding-statistics/statistics-by-subject-area/population/population-composition/population-statistics/.

[19] Bendtsen M., Bendtsen P., Henriksson H., Henriksson P., Müssener U., Thomas K., Löf M. (2020). The mobile health multiple lifestyle behavior interventions across the Lifespan (MoBILE) research program: Protocol for development, evaluation, and implementation. J. Med. Internet Res..

[20] Patton M.Q. (2015). Qualitative Research & Evaluation Methods.

[21] Braun V., Clarke V. (2006). Using thematic analysis in psychology. Qual. Res. Psychol..

[22] Tong A., Sainsbury P., Craig J. (2007). Consolidated criteria for reporting qualitative research (COREQ): A 32-item checklist for interviews and focus groups. Int. J. Qual. Health Care.

[23] Ngongalah L., Rankin J., Rapley T., Odeniyi A., Akhter Z., Heslehurst N. (2018). Dietary and physical activity behaviours in African migrant women living in high income countries: A systematic review and framework synthesis. Nutrients.

[24] Persson G., Mahmud A.J., Hansson E.E., Strandberg E.L. (2014). Somali women’s view of physical activity—A focus group study. BMC Womens Health.

[25] Goodwin L., Hunter B., Jones A. (2018). The midwife–woman relationship in a South Wales community: Experiences of midwives and migrant Pakistani women in early pregnancy. Health Expect..

[26] Tankwanchi A.S., Bowman B., Garrison M., Larson H., Wiysonge C.S. (2021). Vaccine hesitancy in migrant communities: A rapid review of latest evidence. Curr. Opin. Immunol..

[27] Merry L., Villadsen S.F., Sicard V., Lewis-Hibbert N. (2020). Transnationalism and care of migrant families during pregnancy, postpartum and early-childhood: An integrative review. BMC Health Serv. Res..

[28] Kasper A., Mohwinkel L., Nowak A.C., Kolip P. (2022). Maternal health care for refugee women—A qualitative review. Midwifery.

[29] Mårtensson L., Lytsy P., Westerling R., Wångdahl J. (2020). Experiences and needs concerning health related information for newly arrived refugees in Sweden. BMC Public Health.

[30] Henriksson H., Alexandrou C., Henriksson P., Henström M., Bendtsen M., Thomas K., Müssener U., Nilsen P., Löf M. (2020). MINISTOP 2.0: A smartphone app integrated in primary child health care to promote healthy diet and physical activity behaviours and prevent obesity in preschool-aged children: Protocol for a hybrid design effectiveness-implementation study. BMC Public Health.

[31] Alexandrou C., Müssener U., Thomas K., Henriksson H., Löf M. (2021). Adapting a parental support app to promote healthy diet and physical activity behaviors (MINISTOP) for a multi-ethnic setting: A qualitative study on the needs and preferences of parents and nurses within Swedish child health care. Nutrients.

[32] Moser A., Korstjens I. (2018). Series: Practical guidance to qualitative research. Part 3: Sampling, data collection and analysis. Eur. J. Gen. Pract..

[33] Korstjens I., Moser A. (2018). Series: Practical guidance to qualitative research. Part 4: Trustworthiness and Series: Practical guidance to qualitative research. Part 4: Trustworthiness and publishing. Eur. J. Gen. Pract..

[34] The World Health Organization Diagnostic Criteria and Classification of Hyperglycaemia First Detected in Pregnancy. https://www.who.int/publications/i/item/WHO-NMH-MND-13.2.

[35] Statistics Sweden ICT Usage in Households and by Individuals 2020. https://www.scb.se/en/finding-statistics/statistics-by-subject-area/living-conditions/living-conditions/ict-usage-in-households-and-by-individuals/pong/publications/ict-usage-in-households-and-by-individuals-2020/.

